# Differential CRH expression level determines efficiency of Cre- and Flp-dependent recombination

**DOI:** 10.3389/fnins.2023.1163462

**Published:** 2023-08-03

**Authors:** Chen Zhao, Clemens Ries, Ying Du, Jingwei Zhang, Kenji Sakimura, Keiichi Itoi, Jan M. Deussing

**Affiliations:** ^1^Molecular Neurogenetics, Max Planck Institute of Psychiatry, Munich, Germany; ^2^Department of Animal Model Development, Brain Research Institute, Niigata University, Niigata, Japan; ^3^Super-Network Brain Physiology, Graduate School of Life Sciences, Tohoku University, Sendai, Japan

**Keywords:** corticotropin-releasing hormone, CRH, CRF, Cre, Flp, reporter, stress

## Abstract

Corticotropin-releasing hormone expressing (CRH^+^) neurons are distributed throughout the brain and play a crucial role in shaping the stress responses. Mouse models expressing site-specific recombinases (SSRs) or reporter genes are important tools providing genetic access to defined cell types and have been widely used to address CRH^+^ neurons and connected brain circuits. Here, we investigated a recently generated *CRH-FlpO* driver line expanding the CRH system-related tool box. We directly compared it to a previously established and widely used *CRH-Cre* line with respect to the *FlpO* expression pattern and recombination efficiency. In the brain, *FlpO* mRNA distribution fully recapitulates the expression pattern of endogenous *Crh*. Combining both *Crh* locus driven SSRs driver lines with appropriate reporters revealed an overall coherence of respective spatial patterns of reporter gene activation validating *CRH-FlpO* mice as a valuable tool complementing existing *CRH-Cre* and reporter lines. However, a substantially lower number of reporter-expressing neurons was discerned in *CRH-FlpO* mice. Using an additional CRH reporter mouse line (*CRH-Venus*) and a mouse line allowing for conversion of Cre into FlpO activity (*CAG-LSL-FlpO*) in combination with intersectional and subtractive mouse genetic approaches, we were able to demonstrate that the reduced number of tdTomato reporter expressing CRH^+^ neurons can be ascribed to the lower recombination efficiency of FlpO compared to Cre recombinase. This discrepancy particularly manifests under conditions of low CRH expression and can be overcome by utilizing homozygous *CRH-FlpO* mice. These findings have direct experimental implications which have to be carefully considered when targeting CRH^+^ neurons using *CRH-FlpO* mice. However, the lower FlpO-dependent recombination efficiency also entails advantages as it provides a broader dynamic range of expression allowing for the visualization of cells showing stress-induced CRH expression which is not detectable in highly sensitive *CRH-Cre* mice as Cre-mediated recombination has largely been completed in all cells generally possessing the capacity to express CRH. These findings underscore the importance of a comprehensive evaluation of novel SSR driver lines prior to their application.

## 1. Introduction

Genetically engineered mouse models (GEMMs) are potent tools used in neuroscience research and crucial for understanding mechanisms underlying neurological and psychiatric disorders. *Cre-loxP* and *Flp-FRT* are site-specific recombinase (SSR) systems widely used in GEMMs. The combination of *Cre-/Flp*-expressing mouse strains with recombinase-dependent reporters or effectors has proven as a versatile and efficient approach offering a myriad of options ranging from manipulation of gene expression and cell activity to visualization of molecules, cells and circuits (Deussing, 2013; Luo et al., 2018; Arias et al., 2022).

Corticotropin-releasing hormone (CRH; also known as corticotropin-releasing factor, CRF) is a neuropeptide playing a critical role in different aspects of the body’s stress response. CRH in the paraventricular nucleus of the hypothalamus (PVN) controls the neuroendocrine response by activating the hypothalamic–pituitary–adrenal (HPA) axis and regulating adrenocorticotropic hormone (ACTH) secretion from the pituitary which, in turn, triggers the synthesis and release of glucocorticoids from the adrenal glands as ultimate effectors of the stress response (Ulrich-Lai and Herman, 2009). Parvocellular CRH neurons in the PVN are localized at the apex of the HPA axis and represent a specialized subpopulation of excitatory CRH neurons co-expressing the vesicular glutamate transporter 2 (VGLUT2) (Dabrowska et al., 2013; Romanov et al., 2017; Dedic et al., 2018b).

Beyond the PVN, CRH is widely expressed throughout the brain (Peng et al., 2017). Here, in contrast to the PVN, CRH is predominantly found in inhibitory neurons expressing GABAergic markers, such as glutamic acid decarboxylase (also known as glutamate decarboxylase) 65 and 67 (GAD65/GAD67) (Romanov et al., 2017; Dedic et al., 2018b, 2019). Inhibitory CRH neurons show a high degree of morphological and physiological diversity (Wang et al., 2021). For instance, in the cortex, CRH neurons co-express markers of different subpopulations of GABAergic neurons, such as vasoactive intestinal peptide (VIP), calretinin (CR), cholecystokinin (CCK), and somatostatin (SOM), but not parvalbumin (PV) or neuropeptide Y (NPY) (Kubota et al., 2011). In the hippocampus, CRH neurons were found to be positive for PV, CR, and CCK but not for calbindin (CB) or SOM (Gunn et al., 2019). In subregions of the extended amygdala encompassing the central amygdala (CeA), the lateral part of the interstitial nucleus of the posterior limb of the anterior commissure (IPACL) and the bed nucleus of the stria terminalis (BNST), CRH neurons again express a diverse and region-specific set of GABAergic markers including protein kinase C δ (PKCδ), SOM, neurokinin B (NKB), neurotensin (NTS) or CB (Sanford et al., 2017; Dedic et al., 2018b; Chang et al., 2022; Soden et al., 2022). Cortical and hippocampal CRH neurons possess typical features of interneurons. The extended amygdala, however, harbors a large fraction of CRH neurons representing GABAergic long-range projection neurons which co-express calcium/calmodulin-dependent protein kinase 2α (CAMK2A) and innervate midbrain structures, such as the ventral tegmental area and the substantia nigra (Dedic et al., 2018b; Chang et al., 2022; Soden et al., 2022). The heterogeneity of neuronal CRH populations suggests a unique role of these neurons in the regulation of responses to salient stimuli in different brain regions. Accordingly, the CRH system has been shown to bidirectionally modulate anxiety- and stress-related behaviors in dependence of its localization and prior stress experience (Refojo et al., 2011; Lemos et al., 2012; Dedic et al., 2018b).

As a neuropeptide, CRH is sorted into large dense core vesicles and immediately transported to be stored at its release sites, making reliable detection of CRH^+^ soma somewhat difficult. Moreover, it is of note that even with the most efficient antiserum raised against CRH more than 4 decades ago in the Vale lab (Bloom et al., 1982), the accessibility of CRH by immunohistochemistry is largely unsatisfying (Itoi et al., 2014). Hence, extensive efforts have been undertaken in the past years to gain genetic access to CRH^+^ cells by developing respective reporter and SSR mouse lines (Table 1). Different strategies have been applied, ranging from classic transgenic approaches using small promoter fragments to large bacterial artificial chromosome (BAC)-based constructs and knock-in approaches. The first transgenic β-galactosidase reporter mice, carrying a *lacZ* construct flanked by 8.7 kb of *Crh* promoter region, already revealed difficulties in fully recapitulating the endogenous CRH expression pattern (Keegan et al., 1994). This has been further corroborated by some of the subsequently generated transgenic reporter or Cre driver lines revealing limitations in their use because of ectopic expression and/or failure of expression (Alon et al., 2009; Martin et al., 2010; Sarkar et al., 2011; Chen et al., 2015; Korecki et al., 2019). These observations underscore the utter importance to extensively characterize SSR mouse models to validate spatial and temporal expression patterns as a prerequisite for a meaningful interpretation of studies using these GEMMs (Heffner et al., 2012; Luo et al., 2020). In this regard, knock-in strategies, also in the case of CRH, have proven their superiority as reflected by reliable and reproducible reporter and Cre expression (Taniguchi et al., 2011; Itoi et al., 2014; Krashes et al., 2014; Kono et al., 2017). Accordingly, *CRH-Cre* knock-in mice have been widely applied to explore the physiology of CRH neurons in the central nervous system (Krashes et al., 2014; Marcinkiewcz et al., 2016; Fadok et al., 2017; Sanford et al., 2017; Dedic et al., 2018b; Wang et al., 2021; Chang et al., 2022).

**Table 1:** Summary of CRH-related Cre/Flp-driver and reporter mouse lines.

Mouse lines^*^	MGI ID	Description	References
CRH-Cre lines			
CRFp3.0Cre	MGI:4457114	Transgenic – random insertion in mouse genome	Martin et al. (2010)
CRH-Cre(KN282)	MGI:4366795	BAC transgenic – random insertion in mouse genome	Sarkar et al. (2011)
*CRH-ires-Cre*	MGI:4452089	Knock-in – in the endogenous *Crh* locus	Taniguchi et al. (2011)
*Crh-IRES-Cre*	MGI:5559540	Knock-in – in the endogenous *Crh* locus	Krashes et al. (2014)
*CRF-iCre*	MGI:5707348	Knock-in – in the endogenous *Crh* locus	Itoi et al. (2014)
CRH-icreERT2	MGI:5568222	BAC transgenic – insertion in the *Hprt* locus	Korecki et al. (2019)
CRH-Flp line			
*Crh-IRES-FlpO*	MGI:6116854	Knock-in – in the endogenous *Crh* locus	Salimando et al. (2020)
CRH reporter lines			
8.7 CRH β-gal	n/a	Transgenic – random insertion in mouse genome	Keegan et al. (1994)
*CRH-GFP*	MGI:5586654	BAC transgenic – random insertion in mouse genome	Alon et al. (2009)
*CRF-Venus*	MGI:5707346	Knock-in – in the endogenous *Crh* locus	Itoi et al. (2014)
*CRF-Venus∆Neo*	MGI:6144041	Knock-in – in the endogenous *Crh* locus	Kono et al. (2017)

^*^Names of mouse lines adhere to the nomenclature used in the indicated references. CRH/CRF, corticotropin releasing hormone/factor; MGI, mouse genome informatics; n/a, not available.

The *Flp-FRT* system was initially introduced to remove selection markers from *Cre-loxP*-dependent conditional alleles (Rodríguez et al., 2000) but with the concerted improvement of its recombination efficiency (Buchholz et al., 1998; Raymond and Soriano, 2007) the *Flp-FRT* system has developed into a powerful alternative SSR which, in combination with the *Cre-loxP* system, allows for parallel, intersectional or subtractive approaches. Only recently, a first *CRH-FlpO* mouse line was established, thus expanding the CRH system-related tool box (Salimando et al., 2020) and providing alternative access to CRH neurons, which is highly demanded considering their enormous diversity. However, a systematic characterization of this novel mouse line has not been reported so far which to some extent limits its immediate use. In particular, considering repeated accounts on the diversity of reporter expression conveyed by previously established *CRH-Cre* lines demands a comprehensive assessment of the recombination pattern in this novel *CRH-FlpO* mouse line (Chen et al., 2015; Dedic et al., 2018a).

Here, we characterized *CRH-FlpO* mice and directly compared their recombination properties throughout the brain with a well-established *CRH-Cre* mouse line as a reference (Taniguchi et al., 2011) using 9 different reporter mouse models. We focused our evaluation of SSR-activated reporter gene expression patterns on brain regions well-known to possess CRH^+^ neurons involved in the neuroendocrine and behavioral stress response. Our analysis revealed similarities but also discrepancies between *CRH-FlpO* and *CRH-Cre* mice that have direct implications for the use of SSRs driven by the *Crh* locus.

## 2. Materials and methods

### 2.1. Animals

All animal experiments were conducted with the approval of and in accordance with the Guide of the Care and Use of Laboratory Animals of the Government of Upper Bavaria, Germany. Mice were group- housed under standard lab conditions (22 ± 1°C, 55 ± 5% humidity) and maintained under a 12 h light–dark cycle with food and water *ad libitum*. All experiments were conducted with adult male or female mice (age: 2–5 months). For each mouse line 3–7 animals were analyzed of which representative images are included in the figures.

The following transgenic mouse lines were used in this study: *CRH-Cre* (*Crh^tm1(cre)Zjh^*, Jackson Laboratory stock no. 012704) (Taniguchi et al., 2011), *CRH-FlpO* (*Crh^tm1.1(flpo)Bsab^*, Jackson Laboratory stock no. 031559; Supplementary Material, provided by Bernado Sabatini and Caiying Guo) (Salimando et al., 2020), *CRH-Venus* (*Crh^tm1.1Ksak^*, RIKEN BRC stock no. BRC09893) (Kono et al., 2017), *Ai9* (*Gt(ROSA)26Sor^tm9(CAG-tdTomato)Hze^*, Jackson Laboratory stock no. 007909) (Madisen et al., 2010), *Ai65* (*Gt(ROSA)26Sor^tm65.1(CAG-tdTomato)Hze^*, Jackson Laboratory stock no. 021875) (Madisen et al., 2015) and *CAG-LSL-FlpO* (*Gt(ROSA)26Sor^tm5(CAG-flpo)Zjh^*, Jackson Laboratory stock no. 028584) (He et al., 2016). A pure Flp reporter line was generated by breeding the intersectional *Ai65* reporter to a *Deleter-Cre* line removing the *loxP*-flanked transcriptional terminator (STOP). This novel mouse line, reporting only Flp recombinase activity, was designated as *Ai65F*. In this study, the following double and triple transgenic lines were generated by cross-breeding of single transgenic lines: *CRH-Cre::Ai9*, *CRH-FlpO::Ai65F*, *CRH-Venus::CRH-Cre::Ai9*, *CRH-Venus::CRH-FlpO::Ai65F, CRH-Cre::CRH-FlpO::Ai65*, *CRH-Cre::CRH-FlpO::Ai9*, *CRH-Cre::CAG-LSL-FlpO::Ai65* and *CRH-Cre::CAG-LSL-FlpO::Ai9*. All transgenic alleles involving the *Rosa26* locus were heterozygous. Detailed breeding schemes, primer sequences and protocols for genotyping are available upon request.

### 2.2. Preparation of brain sections

*Vibratome sections*: mice were sacrificed by an overdose of isoflurane and transcardially perfused with 20 mL 0.1 M PBS (4°C) supplemented with heparin and subsequently with 20 mL of 4% paraformaldehyde (PFA) in 0.1 M PBS (4°C). After recovery, brains were post-fixed overnight in 4% PFA at 4°C and coronal 50 μm sections were prepared using a vibratome (Microm HM 650 V, Thermo-Fisher Scientific). *Cryosections*: perfused and dissected brains were post-fixed in 4% PFA for 6 h. After 48 h in 30% sucrose in 0.1 M PBS at 4°C, brains were snap frozen on dry ice and 40 μm sections were prepared using a cryostat (Microm, Walldorf, Germany). All sections were stored at −20°C in cryopreservation solution (25% glycerol, 25% ethylene glycol, 50% 0.1 M PBS, pH 7.4) until further use.

### 2.3. Immunohistochemistry

Vibratome sections were blocked with 10% normal goat serum (NGS) and 0.15% Triton in PBS for 30 min at RT and then washed in 0.1 M PBS for 3 × 5 min. Subsequently, sections were incubated with anti-GFP primary antibody (chicken polyclonal antibody; 1:500; abcam) diluted in 0.15% NGS and 0.15% Triton in PBS overnight at 4°C. After another washing step, sections were incubated with an Alexa Fluor 488 secondary antibody (goat anti-chicken IgG; 1:250; Invitrogen) while shaking for 2 h at RT. Following 3 more washing steps, sections were mounted on glass slides (epredia) with Fluoromount-G (SouthernBiotech).

### 2.4. *In situ* hybridization

Freshly dissected brains were snap frozen on dry ice and stored at −80°C until further use. Brains were sectioned coronally at 20 μm using a cryostat. Sections were mounted onto SuperFrost slides and kept at −80°C until further use. ISH was performed as previously described (Dedic et al., 2018b). The following FlpO-specific riboprobe was used: bp 311–641 of MH493814.

### 2.5. Acute restraint stress

To test the capacity of the *CRH-FlpO* mouse line to induce reporter gene expression in CRH^+^ cells following stress exposure, *CRH-FlpO::Ai65F* mice were exposed to acute restraint stress. On the day of the experiment, animals were placed for 15 min in a 50 ml conical tube with the bottom removed. Animals were sacrificed 1 week later and brains were used for the assessment of stress-induced expression of tdTomato.

### 2.6. Image acquisition

Vibratome sections and cryosections were imaged using a slidescanner or confocal microscope. *Slidescanner*: overviews of whole brain sections and region-specific scans were performed with an Olympus VS120 Slide Scanner. Image capturing and initial processing was conducted with the Olympus VS Software. Briefly, overview images of the whole slide were acquired in the batch scanning mode. Subsequently, region-specific scans were taken with the 20×, whole sections images with the 10× objective. The scan region was adjusted to the size of the region. Exposure time and focus were set to automated mode. Sections were acquired with the Cy3 (TdTomato) channel or the FITC (GFP) channel. *Confocal microscopy*: for higher resolution, confocal images of regions of interest were acquired with a Zeiss Axioplan2 fluorescence microscope using Axio Vision 4.5 software. Fluorescently labeled cells were excited at 488 nm (GFP Alexa-488) and 559 nm (tdTomato, Alexa-594), respectively, using a 20× objective. After acquisition in virtual slide (.VSI file) or Carl Zeiss (.CZI) format, images were extracted and saved as Tagged Image File Format (.tiff) for further processing. Images were analyzed using Fiji^1^ and Inkscape (version 1.2.2.).

## 3. Results

### 3.1. FlpO expression pattern in *CRH-FlpO* mice recapitulates endogenous CRH expression

**Figure 1:**
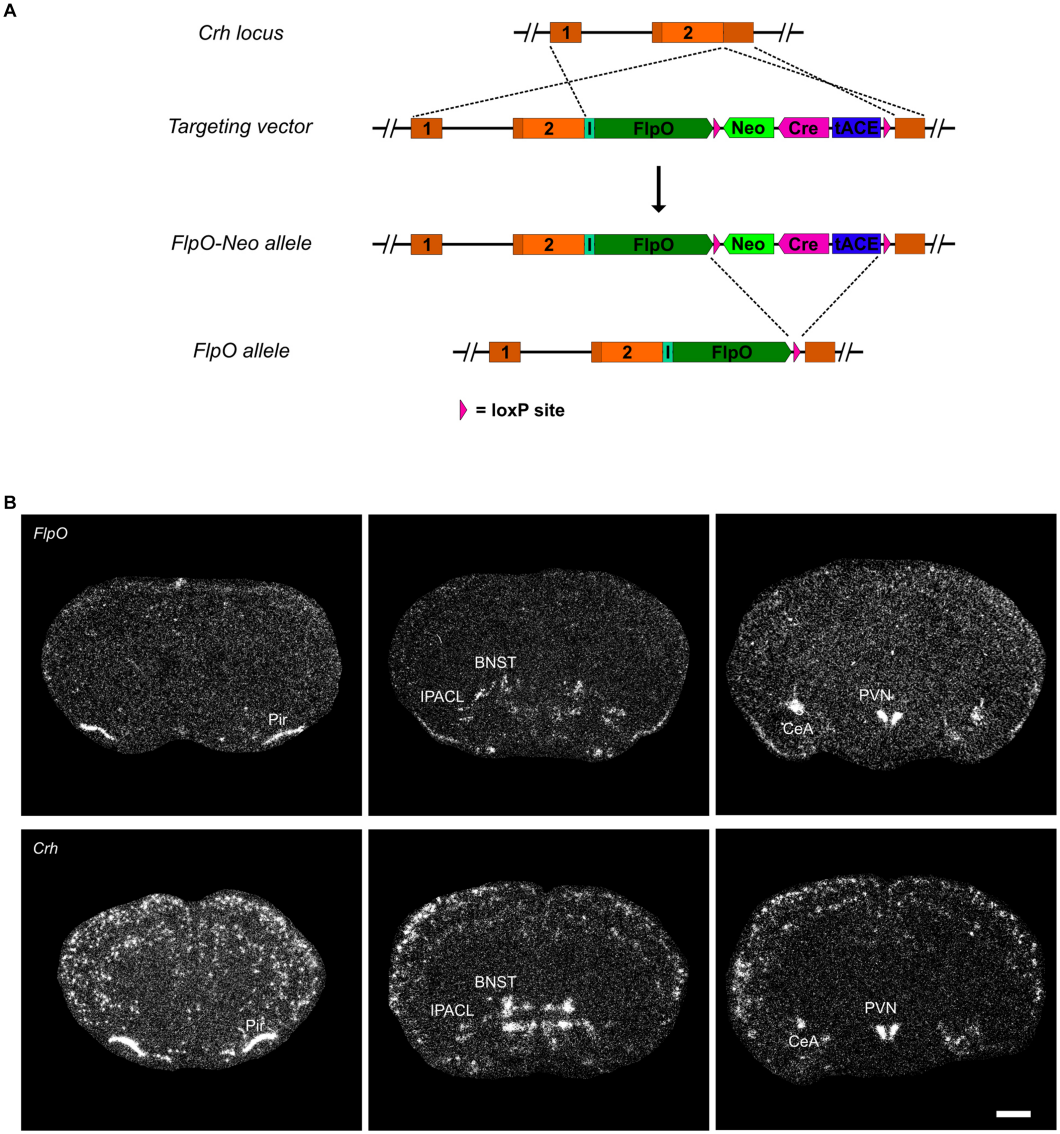
FlpO expression pattern in *CRH-FlpO* mice recapitulates endogenous CRH expression. **(A)** Scheme illustrating the generation of *CRH-FlpO* mice by homologous recombination-mediated gene targeting. **(B)** *In situ* hybridization (ISH) on coronal brain sections of heterozygous (*Crh^+/FlpO^*) (*n* = 3) and wild-type (*Crh^+/+^*) *CRH-FlpO* (*n* = 3) mice using *FlpO*- and *Crh*-specific riboprobes showing comparable expression patterns, e.g., the in piriform cortex (Pir), lateral part of the interstitial nucleus of the anterior commissure (IPACL), bed nucleus of the stria terminalis (BNST), central amygdala (CeA) and paraventricular nucleus of the hypothalamus (PVN). I, internal ribosomal entry site; *loxP*, *loxP2272* Neo, neomycine resistance gene. Scale bar, 1,000 μm.

*CRH-FlpO* mice were generated by targeted insertion of a FlpO expression cassette into the murine *Crh* locus immediately downstream of the stop codon. FlpO expression driven by the *Crh* locus is conveyed by an internal ribosomal entry site (IRES). As a selection marker, a self-excising neomycin cassette driven by the testis-specific angiotensin converting enzyme promotor (tACE) was used, which removed itself in the male germline leaving a single *loxP2272* site behind (Figure 1A and Supplementary Material). To validate FlpO expression, we used heterozygous *CRH-FlpO* mice and performed an *in situ* hybridization (ISH) against *FlpO*. The spatial pattern of *FlpO* mRNA expression fully recapitulated the expression of endogenous *Crh* as indicated by the detection of distinct signals in all regions of salient CRH expression including the piriform cortex (Pir), IPACL, BNST, CeA, and PVN. The expression of *FlpO* appeared generally weaker, as *CRH-FlpO* mice were only heterozygous (Figure 1B).

### 3.2. Comparison of FlpO- and Cre-activated reporter gene expression

**Figure 2:**
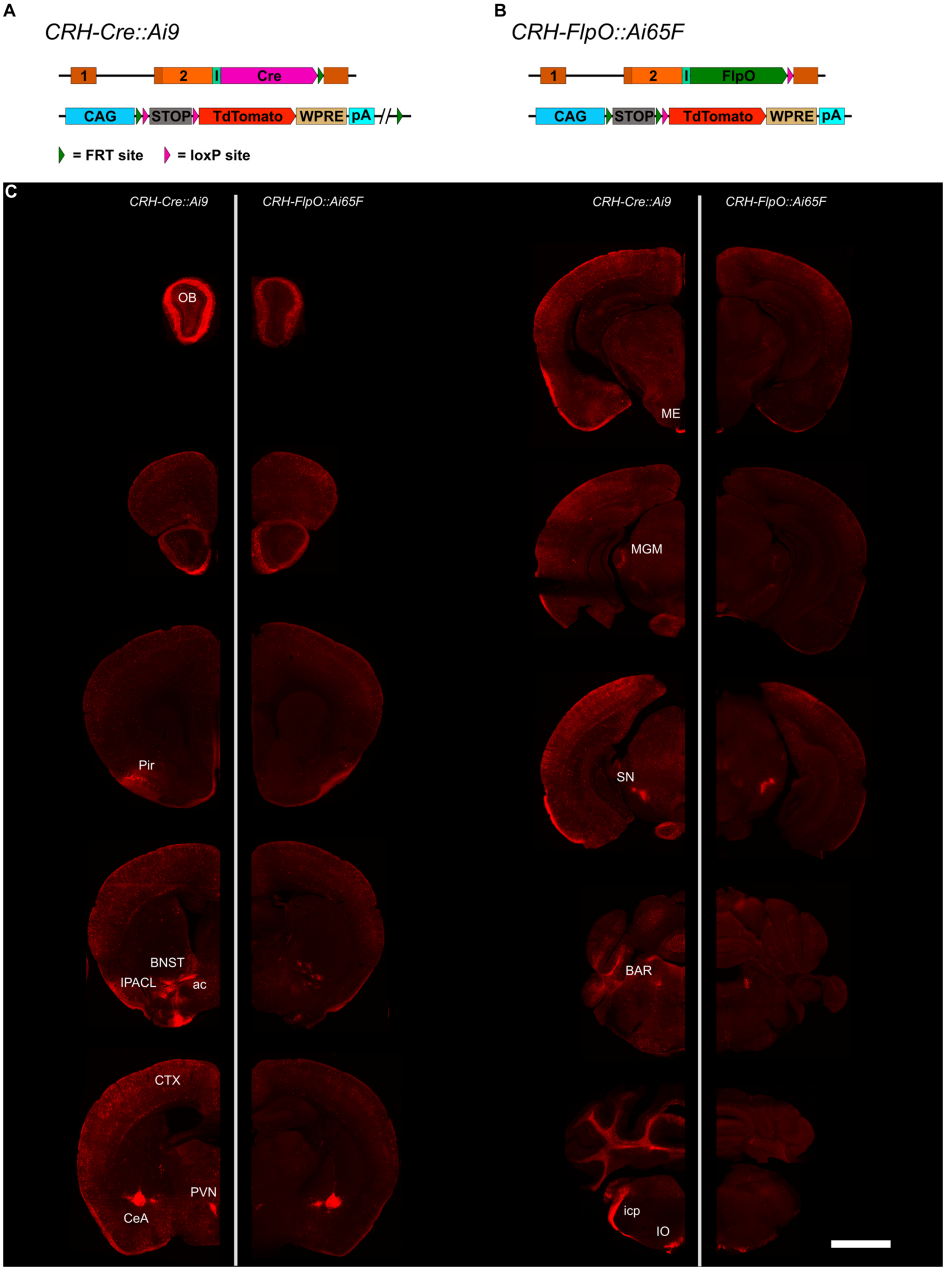
Comparison of the distribution of tdTomato^+^ cells and fibers in the brain of *CRH-Cre::Ai9* and *CRH-FlpO::Ai65F* mice. **(A,B)** Schematic illustration combining photomicrographs of *CRH-Cre::Ai9* (left, *n* = 5) and *CRH-FlpO::Ai65F* (right, *n* = 4) reporter mice. **(C)** Distribution of tdTomato^+^ cells and fibers in *CRH-Cre::Ai9* and *CRH-FlpO::Ai65F* mice. ac, anterior commissure; BAR, Barrington’s nucleus; BNST, bed nucleus of the stria terminalis; CeA, central amygdala; I, internal ribosomal entry site; icp, inferior cerebellar peduncle; IO, inferior olivary nucleus; IPACL, lateral part of the interstitial nucleus of the anterior commissure; ME, median eminence; MGM, medial division of the medial geniculate; OB, olfactory bulb; Pir, piriform cortex; PVN, paraventricular nucleus of the hypothalamus; SN, substantia nigra. WRPE, woodchuck hepatitis virus posttranscriptional regulatory element. Scale bar, 1,000 μm.

To compare the pattern and efficiency of FlpO- and Cre-mediated recombination driven by the *Crh* locus, we bred *CRH-FlpO* and *CRH-Cre* mice to appropriate reporter lines (Figures 2A,B). The reporter alleles in *Ai65F* and *Ai9* mice are ideally suited for direct comparison of FlpO and Cre activity, as both are based on the *Rosa26* locus harboring an identical *CAG* promotor-driven tdTomato expression unit, which is preceded by an *FRT*- and a *loxP*-flanked transcriptional terminator, respectively (Figures 2A,B). Similarly, the *Crh* alleles harboring either FlpO or Cre recombinase were designed in the same way, i.e., the recombinase was integrated immediately downstream of the *Crh* STOP codon and is driven by an IRES.

The tdTomato expression pattern was analyzed throughout the brain of *CRH-FlpO::Ai65F* and *CRH-Cre::Ai9* mice. In both mouse lines, tdTomato-positive neurons were detectable in all brain regions which have previously been reported to endogenously express CRH (Kono et al., 2017; Wang et al., 2021), including the olfactory bulb (OB), cortex (CTX), IPACL, BNST, PVN, CeA, Barrington’s nucleus (BAR) and inferior olivary nucleus (IO) (Figure 2C). Moreover, characteristic tdTomato-labeled CRH^+^ fibers were visible in the anterior commissure (ac) and the inferior cerebellar peduncle (icp), the latter representing climbing fibers originating in the IO and innervating the cerebellum (Ezra-Nevo et al., 2018). In addition, prominent tdTomato-labeled terminals were detectable in the ME and substantia nigra (SN), sites well-known for their massive CRH afferents (Chang et al., 2022). Taken together, our results indicate that the *CRH-FlpO* mice are able to promote site-specific recombination at endogenous CRH expression sites comparable with *CRH-Cre* mice. However, imaging with identical exposure times revealed an overall lower fluorescence intensity detectable throughout brain sections of *CRH-FlpO::Ai65F* compared to *CRH-Cre::Ai9* mice (Figure 2C). This discrepancy in intensity likely originates from a reduced number of tdTomato-labeled soma and fibers present in *CRH-FlpO::Ai65F* mice, an observation which necessitated closer inspection.

### 3.3. FlpO-dependent recombination is inferior to Cre-mediated recombination

**Figure 3:**
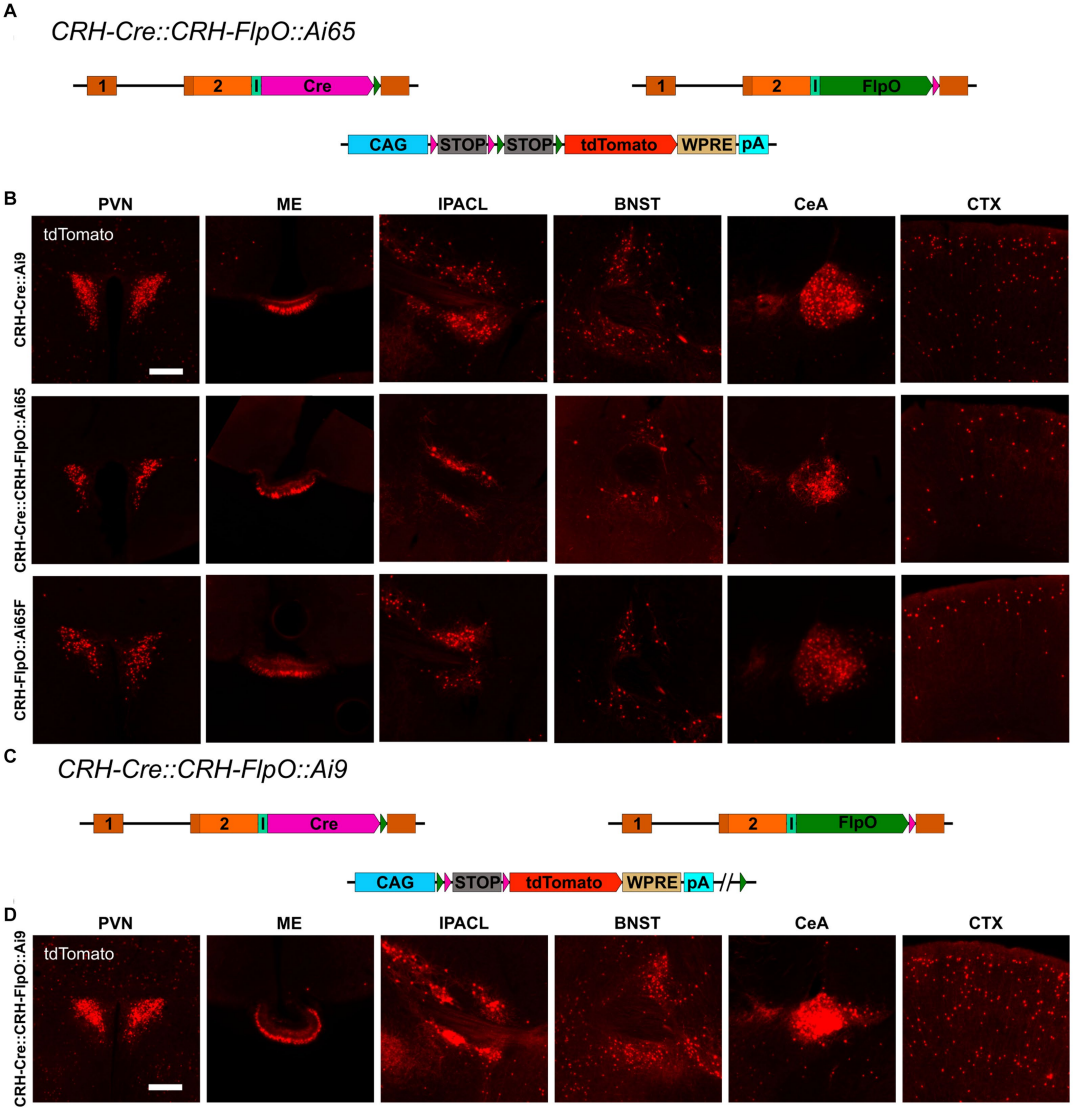
Reporter mice reveal diverging FlpO- and Cre-mediated recombination efficiency. **(A)** Schematic illustration of *CRH-Cre::CRH-FlpO::Ai65 reporter* mice. **(B)** Comparison of the distribution of tdTomato^+^ cells and fibers in regions of interest in *CRH-Cre::Ai9* (*n* = 5), *CRH-Cre::CRH-FlpO::Ai65* (*n* = 4) and *CRH-FlpO::Ai65F* (*n* = 4) mice. **(C)** Schematic illustration of *CRH-Cre::CRH-FlpO::Ai9* reporter mice. **(D)** Distribution of tdTomato^+^ cells and fibers in regions of interest in *CRH-Cre::CRH-FlpO::Ai9* mice (*n* = 6). BNST, bed nucleus of the stria terminalis; CeA, central amygdala; I, internal ribosomal entry site; CTX, cortex; IPACL, lateral part of the interstitial nucleus of the anterior commissure; ME, median eminence; pA, BGH polyA signal; PVN, paraventricular nucleus of the hypothalamus; WRPE, woodchuck hepatitis virus posttranscriptional regulatory element. Scale bars, 200 μm.

To interrogate the discrepancy in tdTomato expression between *CRH-FlpO::Ai65F* and *CRH-Cre::Ai9* mice in more detail, we focused our analysis on CRH-expressing brain structures involved in the neuroendocrine (HPA axis: PVN, ME) and behavioral (extended amygdala: CeA, IPACL, BNST) stress response as well as on the CTX, because of its homogeneous and scattered distribution of CRH^+^ neurons. First, we tested the degree of overlapping Cre and FlpO expression, comparing the occurrence of tdTomato^+^ cells in the intersectional reporter mouse line *CRH-Cre::CRH-FlpO::Ai65* with *CRH-Cre::Ai9* mice (Figure 3A). In all analyzed structures, *CRH-Cre::CRH-FlpO::Ai65* mice showed a markedly reduced number of tdTomato^+^ cells compared to *CRH-Cre::Ai9* mice. Instead, the number of tdTomato^+^ cells was comparable to *CRH-FlpO::Ai65F* mice, suggesting that the FlpO-mediated recombination in CRH^+^ cells was less efficient compared to Cre-mediated recombination driven by the *Crh* locus (Figure 3B).

To rule out the possibility that differences in the genetic composition in which the tdTomato reporter is embedded in *Ai9* vs. *Ai65F* mice was causal for the differential reporting, we used the subtractive capacity of the *Ai9* reporter line. This reporter line still contains the neomycin selection marker and a downstream *FRT* site, which makes the tdTomato expression cassette vulnerable to deletion via Flp recombinase, resulting in a loss of tdTomato expression in cells previously activated by Cre-mediated recombination (Figure 3C). However, the analysis of the PVN, ME, IPACL, BNST, CeA, and CTX in *CRH-Cre::CRH-FlpO::Ai9* mice did not reveal any substantial reduction in tdTomato expression but instead showed similar numbers of tdTomato expressing cells as detected in *CRH-Cre::Ai9* mice (Figure 3D).

Taken together, these data confirm a high degree of co-expression of Cre and FlpO driven from the *Crh* locus but also provide evidence that the Cre recombinase is more efficiently activating reporter gene expression compared to the FlpO recombinase.

### 3.4. Discrepancies between direct and indirect reporting of CRH expression

**Figure 4:**
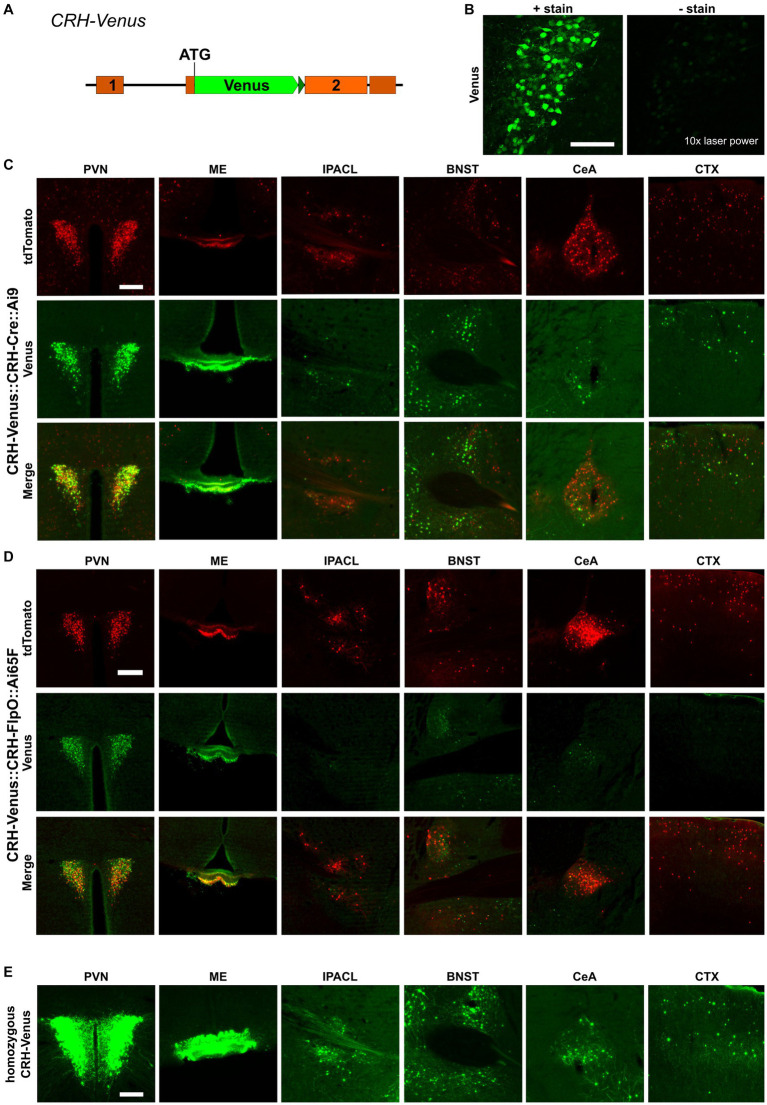
Cre recombinase possesses high recombination efficiency, even at low expression levels. **(A)** Schematic illustration of *CRH-Venus* reporter mice. **(B)** Visualization of Venus^+^ cells with or without GFP antibody staining. Scale bar, 100 μm. **(C)** Comparison of the distribution of tdTomato^+^ and Venus^+^ cells and fibers in regions of interest in *CRH-Venus::CRH-Cre::Ai9* mice (*n* = 3). Scale bar, 200 μm. **(D)** Comparison of the distribution of tdTomato^+^ and Venus^+^ cells and fibers in regions of interest in *CRH-Venus::CRH-FlpO::Ai65F* mice (*n* = 6). Scale bar, 200 μm. **(E)** Distribution of Venus^+^ cells and fibers in regions of interest in homozygous *CRH-Venus* mice (*n* = 3). Scale bar, 200 μm. BNST, bed nucleus of the stria terminalis; CeA, central amygdala; CTX, cortex; IPACL, lateral part of the interstitial nucleus of the anterior commissure; ME, median eminence; PVN, paraventricular nucleus of the hypothalamus.

The recombination efficiency is determined by intrinsic properties of the respective SSR itself and by environmental conditions, e.g., the accessibility of recognition sites within the chromatin structure. Another important aspect is the expression, which needs to reach a certain threshold to allow site-specific recombination. To evaluate the influence of the expression level, we used *CRH-Venus* reporter mice in which the fluorescent protein Venus was integrated at the *Crh* start codon. This direct reporter allele readily reports CRH expression and is at the same time a CRH knockout allele (Figure 4A), whereas *CRH-Cre::Ai9* and *CRH-FlpO::Ai65F* mice serve as indirect reporters enabling the identification of CRH^+^ cells which are labeled by accumulating and enduring tdTomato expression subsequent to CRH expression. Generally, the CRH expression level is rather low and does not allow reliable detection of Venus expression without antibody staining (Figure 4B). We generated *CRH-Venus::CRH-Cre::Ai9* and *CRH-Venus::CRH-FlpO::Ai65F* mice to directly compare CRH-Venus expression with Cre/FlpO-induced tdTomato expression, i.e., direct versus indirect reporting. Detailed analysis revealed a largely congruent expression in all regions of interest in both mouse lines (Figures 4C,D), i.e., a significant proportion of tdTomato^+^ cells showed co-expression of Venus. The distribution of Venus^+^ cells in both mouse lines shows a high degree of concordance indicating similar levels of CRH expression. Thus, the differences observed in tdTomato^+^ cells cannot be attributed to general variations in CRH expression in the two mouse lines. Moreover, we observed in *CRH-Venus::CRH-Cre::Ai9* mice an excess of tdTomato^+^ cells compared to Venus expressing cells (Supplementary Figure S1). The detection of tdTomato^+^/Venus^−^ cells suggests that either (i) Cre caused ectopic CRH reporting by tdTomato, (ii) CRH expression remained below a certain threshold, preventing reliable visualization by antibody staining, or (iii) CRH expression occurred only transiently during development but had ceased in adulthood. To evaluate the influence of the Venus expression level on the number of reported neurons, we bred *CRH-Venus* mice to homozygosity, which resulted in a significantly enhanced Venus signal and an increased number of Venus^+^ cells in all regions of interest. This result indicated that tdTomato^+^/Venus^−^ neurons were not a consequence of ectopic Cre expression but rather reflected their low level of CRH expression. Accordingly, the Venus levels of these cells were too low to be detected by antibody staining, whereas the similarly low amount of Cre recombinase was sufficient to activate tdTomato expression (Figure 4E). Of note, a small proportion of Venus^+^ cells did not express tdTomato, which additionally suggests some limitations of *CAG* promoter-based reporters located in the *Rosa26* locus (Figure 4C; Supplementary Figure S1). *CRH-Venus::CRH-FlpO::Ai65F* mice generally displayed less tdTomato^+^/Venus^+^ cells. However, no clear correlation between the detected Venus expression level and expression of tdTomato was observed (Figure 4D).

### 3.5. FlpO recombination efficacy in CRH cells depends on the FlpO expression level

**Figure 5:**
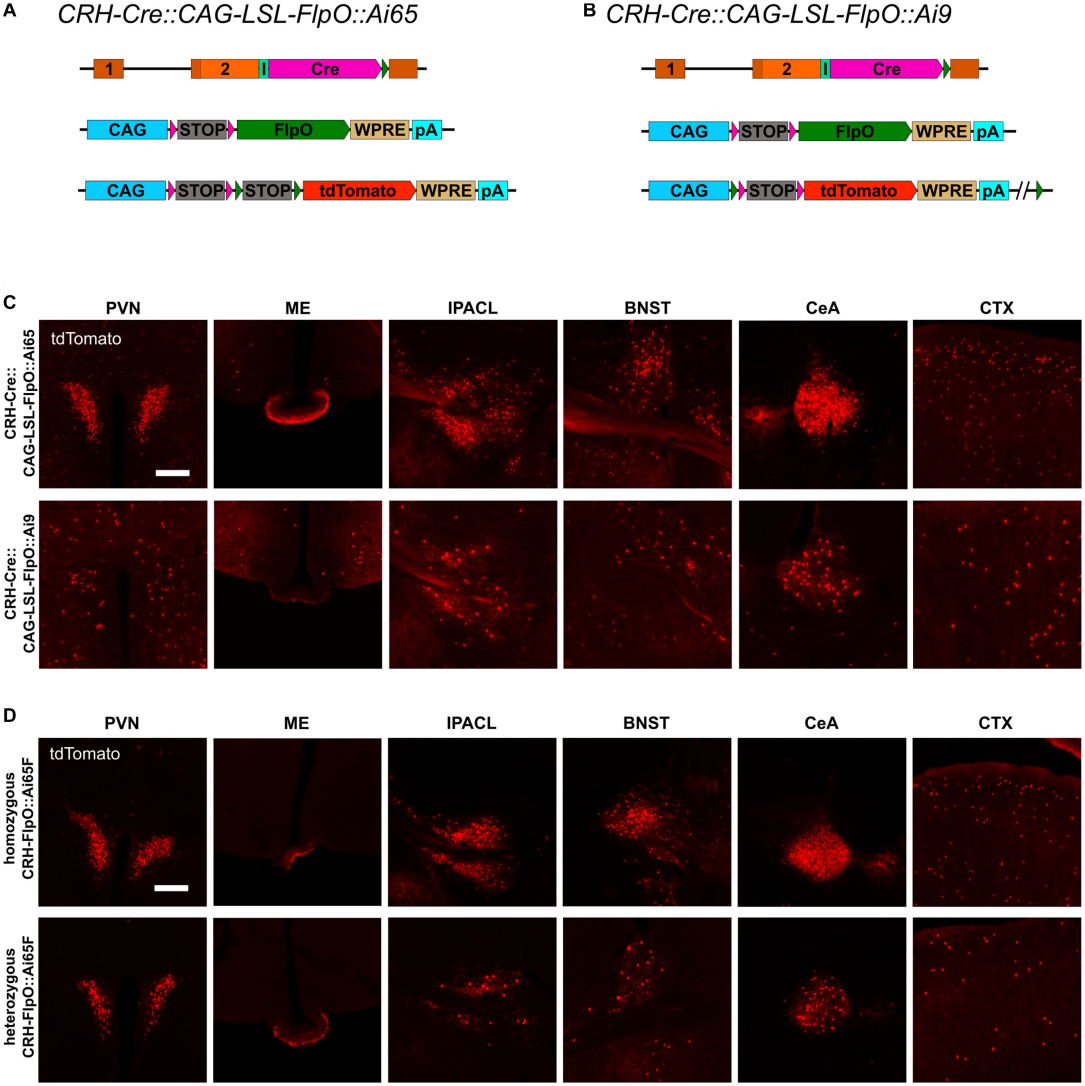
FlpO recombination efficiency depends on strength of driving promoter. **(A,B)** Schematic illustration of *CRH-Cre::CAG-LSL-FlpO::Ai65* and *CRH-Cre::CAG-LSL-FlpO::Ai9* reporter mice. **(C)** Distribution of tdTomato^+^ cells and fibers in regions of interest in *CRH-Cre::CAG-LSL-FlpO::Ai65* (*n* = 3) and *CRH-Cre::CAG-LSL-FlpO::Ai9* (*n* = 7) mice. **(D)** Distribution of tdTomato^+^ cells and fibers in regions of interest in homozygous *CRH-FlpO::Ai65F* mice (*n* = 4) and heterozygous *CRH-FlpO::Ai65F* mice (*n* = 4). BNST, bed nucleus of the stria terminalis; CeA, central amygdala; CTX, cortex; I, internal ribosomal entry site; IPACL, lateral part of the interstitial nucleus of the anterior commissure; ME, median eminence; pA, BGH polyA signal; PVN, paraventricular nucleus of the hypothalamus. Scale bars, 200 μm.

The previous results revealed that the strength of the *Crh* promoter differs strongly between neurons, even in the same region of interest. Therefore, *CAG-LSL-FlpO* mice were used to functionally validate the impact of the expression level on FlpO recombination efficiency. Since the recombination efficiency of a specific recombinase is dependent on its nuclear availability, it is also influenced by its expression level and therefore by the strength of its driving promoter. *CAG-LSL-FlpO* mice allow to convert any Cre activity into FlpO activity, which is then driven by the strong *CAG* promoter. Accordingly, *CRH-Cre::CAG-LSL-FlpO::Ai65* mice express high levels of FlpO in all CRH^+^ cells previously expressing Cre (Figure 5A). Careful analysis of brain regions of interest revealed a considerable increase of tdTomato-expressing CRH neurons throughout the brain resembling the pattern and extent of expression previously observed in *CRH-Ai9* mice (Figure 5C). These findings provide strong evidence for a direct correlation of FlpO recombination efficacy with its expression level in CRH^+^ neurons. Along those lines, and in contrast to *CRH-Cre::CRH-FlpO::Ai9* mice, *CRH-Cre::CAG-LSL-FlpO::Ai9* mice (Figure 5D) showed a substantial reduction of tdTomato-expressing CRH^+^ cells, which was most prominent in the PVN, ME, and CeA (Figure 5C). Along these lines, homozygous mice expressing *FlpO* from both *Crh* alleles, showed a considerable increase in tdTomato^+^ cells compared heterozygous *CRH-FlpO* mice. This result directly proves that the level of FlpO expression determines the efficiency of FlpO recombination in CRH^+^ cells.

Taken together, our results demonstrate that the *FlpO-FRT* system possesses a considerably lower sensitivity compared to the *Cre-loxP* system. Nevertheless, the attenuated sensitivity can be compensated by a higher expression level allowing the *FlpO-FRT* system to reach an efficacy which is comparable to the *Cre-loxP* system.

### 3.6. *CRH-FlpO* mice allow the visualization of stress-induced CRH expression

**Figure 6:**
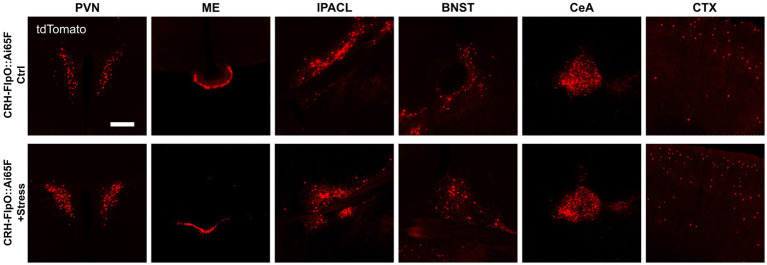
FlpO recombination efficiency is enhanced by acute restraint stress. Comparison of distribution of tdTomato^+^ cells and fibers in regions of interest in stressed and unstressed *CRH-FlpO::Ai65F* mice (each *n* = 5). BNST, bed nucleus of the stria terminalis; CeA, central amygdala; CTX, cortex; I, internal ribosomal entry site; IPACL, lateral part of the interstitial nucleus of the anterior commissure; ME, median eminence; PVN, paraventricular nucleus of the hypothalamus. Scale bar, 200 μm.

While previous studies have demonstrated the faithful reproduction of endogenous CRH expression when combining *CRH-Cre* mice (Taniguchi et al., 2011) with Cre-dependent reporters, such as *CRH-Cre::Ai9*, *CRH-Cre::Ai14* and *CRH-Cre::Ai32* (Chen et al., 2015; Dedic et al., 2018b; Wang et al., 2021; Chang et al., 2022), Walker et al. (2019) reported that there were no significant changes in the number of tdTomato-expressing neurons in *CRH-Cre::Ai14* mice after exposure to stress (Walker et al., 2019). In order to interrogate the suitability of the *CRH-FlpO*-based reporting system for evaluating the response to stress, we exposed *CRH-FlpO::Ai65F* mice to acute restraint stress. Analysis of brain sections revealed a considerable increase in tdTomato-expressing CRH neurons in the investigated brain region which was most prominent in the PVN of stressed compared to unstressed *CRH-FlpO::Ai65F* mice (Figure 6A). These results indicate an upregulation of CRH and concomitant FlpO expression in CRH^+^ neurons following acute stress suggesting that the *CRH-FlpO*-based reporter mouse model provides a more dynamic range of recombination that overcomes the reported limitations of the *CRH-Cre* line (Walker et al., 2019).

## 4. Discussion

CRH^+^ neurons are widely distributed throughout the brain and possess a high diversity with regards to their identity and physiology. The limited accessibility of CRH neurons by antibody-based approaches and particularly the quest to disentangle CRH neuron function has raised increasing interest in CRH-specific SSR and reporter mouse lines. While reliable CRH-specific Cre mouse lines have been available for some time (Taniguchi et al., 2011), only recently, the development of a novel *CRH-FlpO* line (Salimando et al., 2020) has opened up additional opportunities to address the CRH system with increasing precision. To meaningfully apply CRH-specific SSR mouse lines, it is utterly important to understand their capacity to recapitulate endogenous CRH expression. In this study, we therefore compared the recently developed *CRH-FlpO* line with endogenous CRH expression and a well-established and widely used *CRH-Cre* line (Taniguchi et al., 2011). We demonstrated that the overall reporting pattern of *CRH-FlpO* mice is very similar to *CRH-Cre* mice. The efficiency of FlpO, however, is strongly correlated with its expression level in the analyzed population of CRH^+^ neurons.

The comparison of *FlpO* expression with endogenous *Crh* expression on mRNA level and the evaluation of tdTomato induction in *CRH-FlpO::Ai65F* compared to *CRH-Cre::Ai9* mice both confirmed that the FlpO expression and recombination pattern strongly align with that of endogenous *Crh*. These results corroborate the high potential of the chosen knock-in strategy to reliably convey properties of the endogenous *Crh* locus to an integrated transgene.

Nevertheless, the overall intensity of FlpO-activated tdTomato fluorescence appeared somewhat decreased in *CRH-FlpO::Ai65F* compared to *CRH-Cre::Ai9* mice, raising the question whether these discrepancies were caused by differences in their reporting capacity in general. The in-depth analysis of CRH-expressing nuclei revealed that, indeed, the number of tdTomato^+^ labeled cells and fibers were lower in FlpO- than in Cre-dependent reporter animals, strengthening the hypothesis of a differential reporting capacity. Although *CRH-Cre::Ai9* and *CRH-FlpO::Ai65* both utilize tdTomato as a reporter in combination with the *CAG* promoter followed by an identical floxed STOP cassette, the genetic composition in which the tdTomato reporter is embedded differs slightly between the *Ai9* and *Ai65F* lines (Madisen et al., 2010, 2015). Thus, we wondered whether the *Ai65F* reporter, rather than the FlpO recombinase itself, was responsible for the observed differences. Unexpectedly, the intersectional reporter mice *CRH-Cre::CRH-FlpO::Ai65* and *CRH-Cre::CRH-FlpO::Ai9* revealed that FlpO was neither able to report all CRH^+^ cells in the *Ai65*-based model nor did it deplete tdTomato from Cre-activated cells in the *Ai9*-based model. These results imply that not the used *Ai65F* reporter line but FlpO recombination efficiency itself could be the limiting factor. To rule out that the higher cell numbers were not caused by ectopic Cre activity in *CRH-Cre* mice, the *CRH-Venus* mouse line as a direct reporter of CRH expression was used to compare tdTomato and Venus expression in the same individual. It is worth noting that Cre-mediated recombination, resulting in cumulative tdTomato expression, effectively reflects the cellular history of *Crh* gene transcription over the time. In contrast, the Venus^+^ knock-in allele reports a snapshot of the current protein content above a certain detection threshold, providing a glimpse into the present state rather than the developmental timeline. Despite the differences between the direct and indirect reporting systems and the distinct characteristics of the two applied fluorescent proteins, such as their disparate fluorescent protein lifetimes (Sarkar et al., 2009; Drobizhev et al., 2011), the comparable number of Venus^+^ cells observed in the analyzed brain regions of *CRH-Venus::CRH-Cre::Ai9* and *CRH-Venus::CRH-FlpO::Ai65F* mice suggests that there is no variation in CRH expression between the two mouse lines. Accordingly, this indicates that the disparity of tdTomato^+^ cells in *CRH-Cre::Ai9* and *CRH-FlpO::Ai65F* mice may be attributed to the inherent properties of these two recombinases. Indirect reporting via Cre recombinase-mediated activation of tdTomato showed a high degree of co-localization with Venus expression. Interestingly, also a substantial number of tdTomato^+^/Venus^−^ cells were detectable, which left open the possibility that tdTomato expression in Venus^−^ neurons represent ectopic or legacy expression due to transient activation of CRH and, thus, Cre expression, e.g., during development or in response to experienced stressors, which would not be detected by the Venus reporter. In this regard, homozygous *CRH-Venus* mice were instructive, as we were able to visualize an increased number of Venus^+^ cells in all analyzed brain regions compared to heterozygous *CRH-Venus* mice. This observation suggests that more cells reached the detection threshold, supporting the notion that CRH neurons possess a broad spectrum of expression levels, even within the same brain region. This result additionally confirms that Cre-mediated reporter activation is substantially more sensitive than the detection of Venus via antibody staining, as the tdTomato is driven by the strong and ubiquitously active *CAG* promoter. Seemingly, a few molecules of Cre are sufficient to activate tdTomato expression. Although a compensatory upregulation of CRH, caused by the *de facto* knockout of CRH in homozygous CRH-Venus mice, may also have contributed to the higher number of Venus^+^ neurons, the differences in intensity still confirm a broad diversity of CRH expression levels in respective neurons. In contrast to the PVN, CRH expression in the CeA has been reported to be positively regulated by corticosterone (Makino et al., 1994). Consequentially, CRH inactivation, resulting in low corticosterone levels, should lead to a decreased Venus expression in the CeA. Of note, in our experiments we observed a general increase of Venus expression in all regions analyzed, including the CeA. This observation indicates that homozygosity of the reporter is potentially overriding the hypothesized downregulation of CRH by the low corticosterone levels entailed by the CRH knockout.

These results suggest that, if FlpO-mediated recombination, and therefore reporter efficacy, depends on the expression level of the driving promoter, an enhanced expression should be able to elevate the number of reported cells. Indeed, the combination of FlpO with the *CAG* promoter, a strong synthetic promoter integrated in the *Rosa26* locus, did not only increase reporting efficacy in the *CRH-Cre::CAG-LSL-FlpO::Ai65* model to a level comparable to *CRH-Cre::Ai9* mice but was also able to vastly decrease the reporting in the *CRH-Cre::CAG-LSL-FlpO::Ai9* model. These results also demonstrate that the larger distance between the *FRT* sites flanking the tdTomato cassette in the *Ai9* line (3.9 kb) is not limiting FlpO recombination activity in general (Coppoolse et al., 2005; Liu et al., 2013). Ultimately, homozygous *CRH-FlpO::Ai65F* mice, expressing FlpO from both *Crh* alleles, confirmed that the level of FlpO expression is directly correlated with its recombination efficiency. Along these lines, while *CRH-Cre* males show 100% germline recombination when combined with a floxed allele, the respective frequency observed in *CRH-FlpO* males is also significantly lower.

Thermo-instability of yeast-derived Flp-related recombinases has been shown to decrease their recombination efficiency in mammalian cells (Buchholz et al., 1996). Despite previous optimization efforts (Buchholz et al., 1998; Raymond and Soriano, 2007) our results clearly demonstrate a persistent difference in recombination efficiency between Cre and FlpO, which particularly matters in low gene expression conditions. Whether this discrepancy is related to differences in protein thermo-stability or the intrinsic reaction rate of Cre and FlpO, remains to be investigated. To what extent other recombinases, such as Dre, can keep up with Cre recombinase efficiency, requires further examination (Anastassiadis et al., 2009; Karimova et al., 2018). The utilized IRES might further aggravate the situation, as IRES-dependent gene expression of the second gene in a bicistronic construct is up to 10-fold lower than cap-dependent expression of the first gene (Mizuguchi et al., 2000; Martin et al., 2006). Today, 2A-related self-cleaving peptides are used to overcome the drawbacks of IRES (Liu et al., 2017). However, 2A peptides leave a couple of amino acids to the N-terminally located protein. This would not be compatible with CRH maturation which involves proteolytic processing by prohormone convertases at predetermined recognition sites which will be disturbed by remnants of 2A peptides (Hook et al., 2008). In fact, this poses a general difficulty for the expression of neuropeptides using multicistronic expression vectors.

Mouse models targeting the CRH system, particularly those employing Cre-dependent reporters, are widely utilized to study the neurobiology of stress and related circuits. On the one hand, the robust efficacy of Cre recombinase provides a reliable tool for investigating the spatial distribution and connectivity of CRH neurons throughout the brain. On the other hand, the high efficacy of Cre recombination seemingly labels almost all cells which have the potential to express CRH in the course of their lifetime. Consequently, the recruitment of new stress-induced tdTomato^+^ cells in *CRH-Cre::Ai9* cells is very limited (Walker et al., 2019). Our results demonstrate that this particular limitation of *CRH-Cre*-based models can be overcome by using *CRH-FlpO* mice. Following exposure to acute stress, we observed an increased number of tdTomato^+^ cells in *CRH-FlpO::Ai65F* mice. Thus, *de novo* tdTomato expression can serve as a proxy for stress-responsive neurons characterized by the upregulation of the stress peptide CRH. These results highlight the utility of *CRH-FlpO* mice as a valuable tool for functional analysis of the CRH system under both physiological and pathological conditions.

In conclusion, we show a difference in the recombination efficiency of Cre and FlpO in CRH^+^ neurons and demonstrate that the expression level of FlpO contributes to discrepancies in reporter gene activation observed between *CRH-Cre* and *CRH-FlpO* mice. These results have strong implications for future experiments using the investigated mouse lines but also for SSR-based approaches in general. (i) When using and comparing FlpO- and Cre-based reporter systems, the strength of the driving promoter has to be taken into account. (ii) In intersectional approaches, the number of reported co-expressing cells is limited by the efficacy of the FlpO. (iii) In approaches involving knockout or overexpression of a specific target gene, the efficacy is highly dependent on the used SSR. These results are essential to interpret and reconcile divergent scientific discoveries which will ultimately advance our comprehension of the physiological role of CRH in the central nervous system.

## Data availability statement

The raw data supporting the conclusions of this article will be made available by the authors, without undue reservation.

## Ethics statement

The animal study was reviewed and approved by Government of Upper Bavaria, Germany.

## Author contributions

CZ, CR, and YD designed and performed all experiments, analyzed and interpreted data, drafted figures, and wrote the manuscript. JZ performed a subset of experiments and reviewed the manuscript. KS and KI provided materials, edited the manuscript and contributed scientific advice. JD designed the project, supervised all experiments and analyses, edited the manuscript, and provided scientific advice, guidance and support. All authors contributed to the article and approved the submitted version.

## Funding

The project was supported by the Max Planck Society (JD) and the Federal Ministry of Education and Research (FKZ 01KU1901, JD). JZ and YD received funding from the Chinese Scholarship Council. CR received funding from the International Max Planck Research School for Translational Psychiatry and the Gunter Sachs Donation.

## Conflict of interest

The authors declare that the research was conducted in the absence of any commercial or financial relationships that could be construed as a potential conflict of interest.

## Publisher’s note

All claims expressed in this article are solely those of the authors and do not necessarily represent those of their affiliated organizations, or those of the publisher, the editors and the reviewers. Any product that may be evaluated in this article, or claim that may be made by its manufacturer, is not guaranteed or endorsed by the publisher.

## Supplementary material

The Supplementary material for this article can be found online at: https://www.frontiersin.org/articles/10.3389/fnins.2023.1163462/full#supplementary-material

## Footnotes

^1^ http://rsweb.nih.gov/ij/
