# Supplementary material for: Differential CRH expression level determines efficiency of Cre- and Flp-dependent recombination
Source: Front Neurosci. 2023 Aug 3;17:1163462. doi: 10.3389/fnins.2023.1163462 (PMC10434532; doi:10.3389/fnins.2023.1163462)
Supplement: Supplementary file 1 [file Table_1.DOCX]

**Supplementary Material**

**
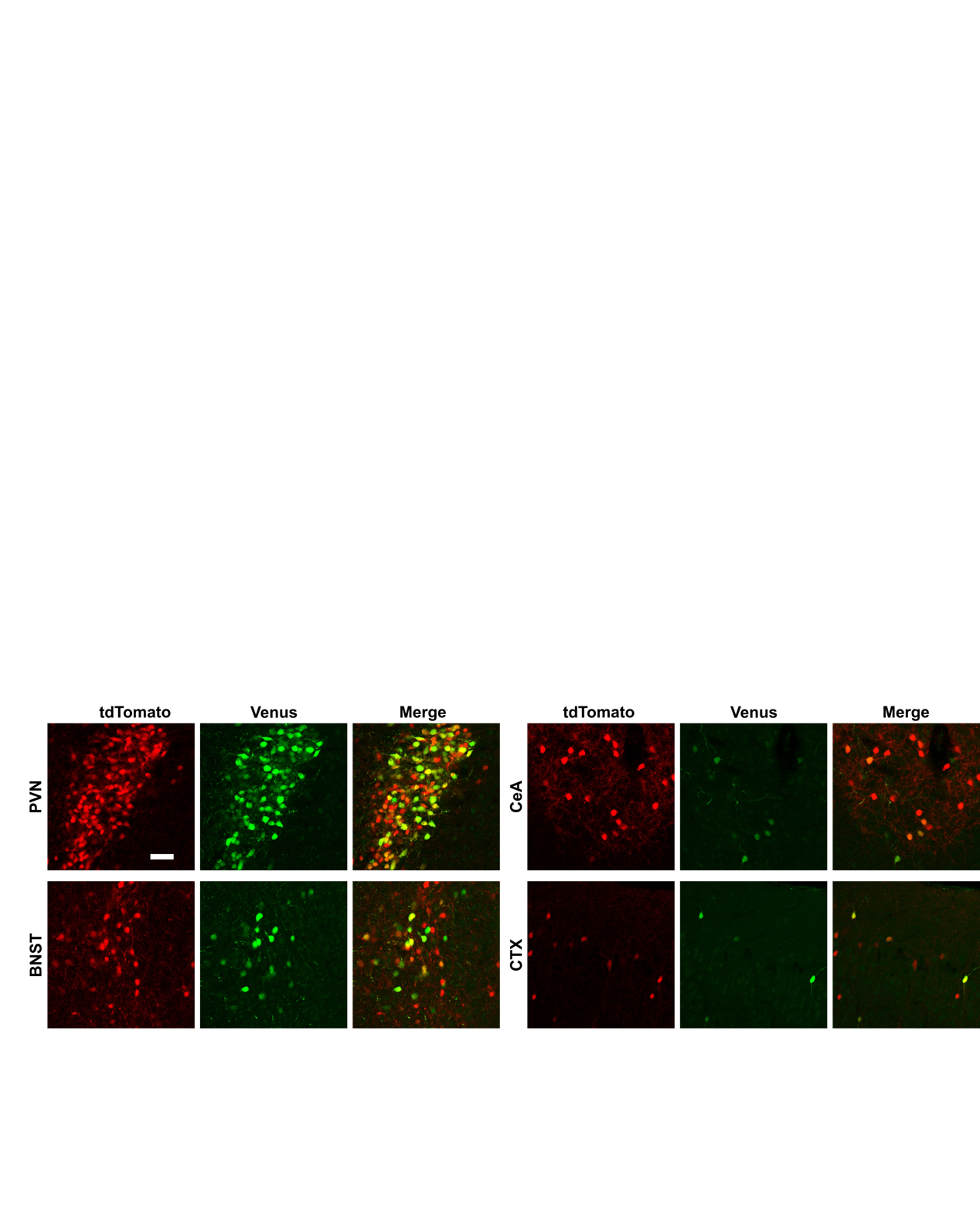
**

**Supplementary Figure S1**

Confocal images of tdTomato^+^ and Venus^+^ cells and ﬁbers in regions of interest in *CRH-Venus::CRH-Cre::Ai9* mice (n = 3). BNST, bed nucleus of the stria terminalis; CeA, central amygdala; CTX, cortex; PVN, paraventricular nucleus of the hypothalamus. Scale bar, 50 μm.

**Supplementary Data**
